# Gun-Shot Wound of the Brain

**Published:** 1855-03

**Authors:** T. S. Smith

**Affiliations:** Murfreesboro’, Tenn.


### CASE OF GUN-SHOT WOUND OF THE BRAIN WITH RECOVERY.

BY T. S. SMITH, M. D., MURFREESBORO', TENN.

I was called on the morning of the 1st of May to see W. J. C. The messenger informed me that he had been shot in the head at 9 o'clock, P. M., April 30th. On my arrival I found him lying on the floor with a blanket thrown over him, where he had lain from the time he was shot until I arrived, which must have been 10 hours. His hair was clotted with blood, and from the statement of those present, he must have lost some 2 or 2½ pounds of blood. He was intoxicated when shot, and was an habitual drunkard. The pulse was 52, intellect wandering, great nausea. I administered a nervous stimulant, and proceeded to examine the wounds. When the hair was removed which hung over his forehead, three wounds were displayed: one a flesh wound over the right eye two inches long, cutting the anterior branch of the temporal artery in its passage; but this had ceased to bleed when I saw him. A second slug (for such were the missiles with which the pistol was charged,) had entered the squamous portion of the temporal bone, and passed out through the frontal bone half an inch to the left of the median line. The dura-mater was plainly visible through the entrance of the slug, and a small probe could be passed to the depth of three inches in the head. The brain was oozing from the exit, and the probe could also be passed to the depth of three inches in this opening. Dr. P. W. Burke was called in counsel, and we agreed to draw together the integument and retain it with adhesive strips to prevent the brain from escaping—a small opening was left for the escape of any fluid that might appear in the wound. He was then placed on a litter and moved to his father's house, distance one mile. By this time some re-action was coming on, and reason somewhat restored; complained of considerable pain in the posterior portion of the head, some subsultus tendinum. One grain of calomel was ordered every hour for six hours, then followed by saline cathartics. Saw him again at 6 P. M. Pulse 100 strokes per minute; skin warm and dry; no nausea, but great thirst—wounds not examined. Ordered neutral mixture every half hour through the night, or as long as the fever continued. Saw him the 2d.—Pulse 72; medicine operated well; skin quite natural; no thirst; intellect good; some subsultus; complaining of pain still in the posterior of the head—dressings removed; brain still oozing out at the exit, but does not appear at the entrance; the dressings were replaced, and the grain doses of calomel again ordered and pushed to moderate ptyalism, with the view if possible to prevent inflammation and promote absorption. 3rd.—Pulse 64; ptyalism pretty well established; brain still disposed to ooze out when the dressings are removed, which continued to be the case until after the seventh day, when the exit was pretty well closed and gave us no further trouble—but the entrance took on fungous growth from the integument, which yielded to the solid stick of nit. arg. and dil. nit. acid alternately. After the seventh day he suffered but little, and had a rapid recovery, without his intellect being at all injured. He lost not less than one or one and a half table spoonfuls of brain. I present the case to the profession without comment.—*Nashville Jour. of Med. and Surg.*

